# Autonomous Reduction and Analysis of 2D Diffraction and Scattering Data

**DOI:** 10.1063/4.0000939

**Published:** 2025-10-27

**Authors:** Anna H Merritt, Wenqian Xu, Olaf Borkiewicz, Miaoqi Chu, Nicholas Schwarz, Brian Toby, James Weng

**Affiliations:** Argonne National Laboratory, Lemont, IL, USA

## Abstract

Current data reduction methods for synchrotron powder diffraction and total scattering experiments involve multiple steps of calibration, masking, and integration of up to thousands of 2D images. The established method of 1D integration helps make this flow of information more human- readable at the cost of azimuthal information such as preferred orientation or the location and number of single crystal diffraction spots. Direct inspection of each 2D image for such information becomes nigh impossible with the amount of data produced per experiment.

The primary goal of this project is to develop a data analysis and reduction pipeline which retains such information while autonomously performing common steps such as masking and integration for the user. These results are then shown to the user in a convenient user interface in real time, allowing for live evaluation and decision making in the middle of an experiment. The first step in retaining the 2D information is classification of outlier pixel clusters into spots and texture arcs. The differences in integrating with and without each set of outliers is shown to the user in a UI alongside the original image and outlier masks to aid in identifying peaks resulting from each type. Statistics on the position and area of these clusters as well as the cosine similarity between subsequent images are also shown in the UI.

